# Sequence and phylogentic analysis of MERS-CoV in Saudi Arabia, 2012–2019

**DOI:** 10.1186/s12985-021-01563-7

**Published:** 2021-04-30

**Authors:** Mohamed A. Farrag, Haitham M. Amer, Rauf Bhat, Fahad N. Almajhdi

**Affiliations:** 1 Department of Botany and Microbiology, College of Science, King Saud University, Riyadh, 11451 Saudi Arabia; 2 Department of Virology, Faculty of Veterinary Medicine, Cairo University, Giza, 12211 Egypt

**Keywords:** Human coronaviruses, Mutation, Viruses, Sequence analysis, Evolution

## Abstract

**Background:**

The Middle East Respiratory Syndrome-related Coronavirus (MERS-CoV) continues to exist in the Middle East sporadically. Thorough investigations of the evolution of human coronaviruses (HCoVs) are urgently required. In the current study, we studied amplified fragments of ORF1a/b, Spike (S) gene, ORF3/4a, and ORF4b of four human MERS-CoV strains for tracking the evolution of MERS-CoV over time.

**Methods:**

RNA isolated from nasopharyngeal aspirate, sputum, and tracheal swabs/aspirates from hospitalized patients with suspected MERS-CoV infection were analyzed for amplification of nine variable genomic fragments. Sequence comparisons were done using different bioinformatics tools available.

**Results:**

Several mutations were identified in ORF1a/b, ORF3/4a and ORF4b, with the highest mutation rates in the S gene. Five codons; 4 in ORF1a and 1 in the S gene, were found to be under selective pressure. Characteristic amino acid changes, potentially hosted and year specific were defined across the S protein and in the receptor-binding domain Phylogenetic analysis using S gene sequence revealed clustering of MERS-CoV strains into three main clades, A, B and C with subdivision of with clade B into B1 to B4.

**Conclusions:**

In conclusion, MERS-CoV appears to continuously evolve. It is recommended that the molecular and pathobiological characteristics of future MERS-CoV strains should be analyzed on regular basis to prevent potential future outbreaks at early phases.

## Background

Coronaviruses (CoVs) have long been known to infect humans and several animal species, causing systemic infections of respiratory airways, intestine, liver, and nervous system [1, 2]. Previously, human CoVs such as HCoV-229E, HCoV-NL63, *HCoV-OC43* and HCoV-HKU1 were known to cause mild and self-limiting respiratory diseases mainly upper respiratory tract infections in the human population. Nevertheless, three outbreaks due to human CoVs have been already witnessed in the last two decades. The first outbreak caused by SARS-CoV-1 originated in China and gradually spread to several countries around the world leading to the death of 774 (9.56%) cases out of 8096 reported to the World Health Organization [3]*.* The causative agent of the second outbreak was identified as MERS-CoV which was originated in Saudi Arabia in September 2012, and spread to the *neighboring* countries and further to other geographically distant regions [4–6].

According to WHO, MERS-CoV outbreak is on-going causing 2519 *laboratory-confirmed* cases till now, of which 866 (34.3%) have died, majority of them in Saudi Arabia. Clinical manifestations of MERS-CoV are similar to those of SARS, nevertheless, MERS-CoV patients mostly develop rapid respiratory failure [5]. The third and the biggest pandemic started in Wuhan, China, in December, 2019. During 1 year since the reporting of the first case, SARS-CoV-2 has affected more than 215 countries with more than 55 million cases. Of these, more than 1.3 million have died with a case fatality rate of more than 2% [7]. Bats are the common reservoir for several beta coronaviruses from which the virus jump to humans through an intermediate hosts [8–10]. However, in case of MERS-CoV, human infections are thought to occur directly from bats or through an intermediate host, most likely dromedary camels [11–13].

MERS-CoV belongs to *the* subfamily *Orthocoronavirinae*, family *Coronaviridae*, and order *Nidovirales*. Viruses of this family are enveloped with polycistronic, non-segmented, positive sense, single-stranded, RNA (~ 30 kbp). According to ICTV, *Orthocoronavirinae* has four genera; *Alphacoronavirus*, *Betacoronavirus*, *Gammacoronavirus*, and *Deltacoronavirus* [14, 15]. Full genome sequence analysis allocated MERS-CoV among betacoronaviruses lineage C, a group that include bat-CoVs [13, 16]. MERS-CoV’s genome was predicted to contain 10 ORFs with the first 5′-three-fourths sequence encodes for RdRp (ORF1a and ORF1b) [16]. These two ORFs are translated via -1 ribosomal frameshifting to yield polyproteins pp1a and pp1b which are processed into 16 nonstructural proteins (nsps) [17, 18]. The last third ORFs are downstream ORF1b and encode for the structural proteins; spike (S), envelop (E), membrane (M) and nucleocapsid (N). Among these proteins, S protein mediates virus entry via attachment to dipeptidyl peptidase 4 (DPP4) [19, 20]. Therefore, S protein is among the highly variable MERS-CoV proteins and hence, a target for phylogenetic studies and designing therapeutic agents.

MERS-CoV transmission, evolution, divergence and animal reservoir are critical issues that should be *resolved for effective control of MERS-CoV and potentially for the current SARS-CoV-2 pandemic*. CoVs are known *for* a high rate of recombination, a feature that enables them to infect several hosts and to fit well in different environmental niches [21–23]. Phylogenetic analysis based on the deduced amino acid sequences of the RdRp domain (ORF1ab, ORF1b) and conserved regions of structural proteins (S, E, N and M) revealed that MERS-CoV is closely related to HCoV-HKU4 and HCoV-HKU5 [14, 24, 25]. Moreover, MERS-CoV shares high sequence identity with bat CoVs from Africa, Europe and America [24, 26]. In the current study, four MERS-CoV isolates of human origin were used for sequence and phylogenetic analysis. Sequence contigs of 6916 nts including ORF1a/b, S, and ORF3/4a were amplified, sequenced and assembled. Sequence analysis was performed and mutations were recorded along the whole sequence contigs. Codons under selective pressure were investigated. Phylogenetic analysis was performed based on the S gene.

## Materials and methods

### Clinical samples

Nasopharyngeal aspirate and/or swab samples were obtained from hospitalized patients with suspected MERS-CoV infection at three different cities in Saudi Arabia: Riyadh, Madinah and Dammam. Sputum and tracheal swabs/aspirates were also considered in few cases. A wide spectrum of clinical symptoms was included as probable signals for MERS-CoV infection including fever, myalgia, cough, dyspnea, aches, abdominal pain, vomiting and diarrhea. Samples were collected and handled by the relevant authorities at the Saudi Ministry of Health (MOH-SA) during December 2014 and January 2015 following the strict biosafety guidelines set by WHO and CDC [7, 27]. Samples were transported in viral transport medium to the regional laboratories of MOH-SA and processed immediately or stored at − 80 °C till use. Informed consents were obtained from the patients or their guardians and the study protocols were prepared to conform to the 1975 Declaration of Helsinki.

### RNA extraction and real-time RT-PCR

Upon receipt, samples were shacked vigorously and equal volumes of sample and external lysis buffer (6 M Guanidine Isothiocyanate, 30% Triton X-100, 100 mM Tris–HCl, 0.01% Bromophenol blue) were mixed in a safety cabinet. Clinical samples were extracted using one of two automatic nucleic acid isolation systems according to their availability in different MOH-SA regional laboratories including: MagNA Pure 96 (Roche Diagnostics, Indianapolis, IN) and QIAsymphony SP (Qiagen, Hilden, Germany). MagNA Pure Compact Nucleic Acid Isolation (Roche) and QIAsymphony RNA (Qiagen) kits were utilized for viral RNA extraction in the corresponding platform according to the manufacturer's instructions. Single negative control of PCR grade water was extracted in parallel for every 12 samples. All samples were screened for MERS-CoV using LightMix® Modular MERS-CoV upE Kit (Roche) using the experimental protocol and reaction setup primarily established by Corman et al. [28]. Positive results were confirmed with LightMix® Modular MERS-CoV ORF1a Kit (Roche). Samples with doubtful results (i.e. positive reactivity in one assay and negative in the other) were re-extracted and tested in triplicates in both UpE and ORF1a assays.

### Generation of sequence data

**Fig. 1:**
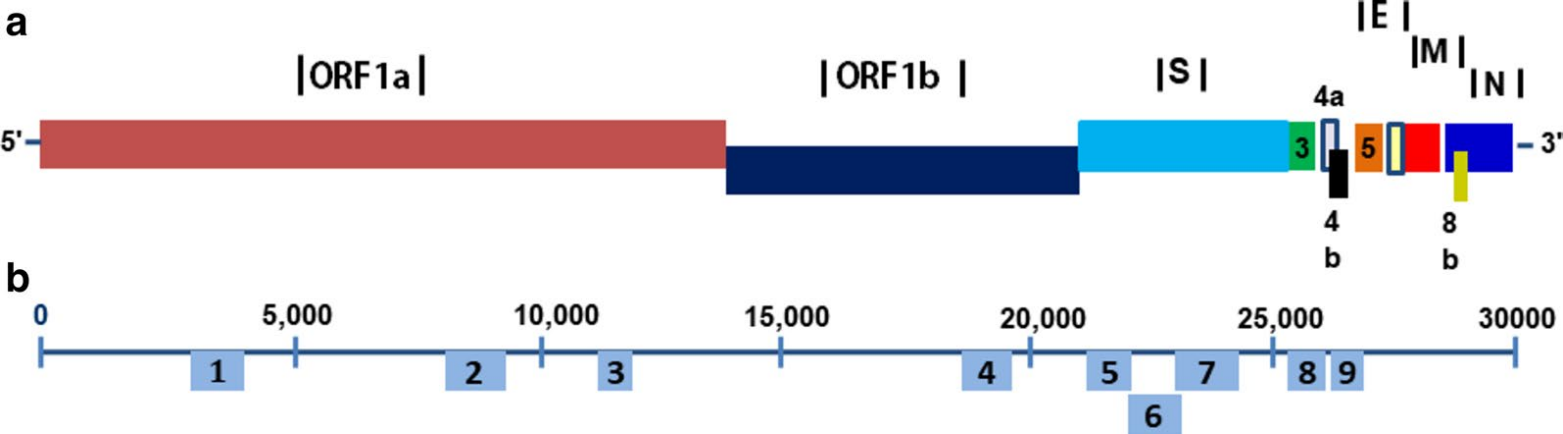
MER-CoV genome depiction and primer design. **a** full length MERS-CoV genome showing different ORFs. **b** location of primers that have been used to amplify the target ORFs

RNA isolated from MERS-CoV positive samples was used for amplification of nine genomic fragments that possess considerable sequence variability among MERS-CoV strains circulating worldwide in man and animals. The nine fragments include four for ORF1a/b, three for S gene, one for ORF3/4a and one for ORF4b (Table 1, Fig. 1). Amplification was performed in a single step RT-PCR using SuperScript® III One-Step RT-PCR System with Platinum® Taq DNA Polymerase (Life Technologies, Carlsbad, CA). The cycling protocol involved: one cycle at 55ºC for 30 min, one cycle at 94ºC for 2 min, 40 cycles of 94ºC for 15 s, 55ºC (58 ºC for fragments 1, 5, 8 and 9) for 30 s and 68ºC for 1 min, and one cycle at 68ºC for 10 min. The amplified fragments were purified using QIAquick PCR purification kit (Qiagen) and were sequenced on both strands using BigDye Terminator version 3.1 sequencing kit on ABI PRISM 3730xl genetic analyzer at GATC Biotech (Cologne, Germany). The sequence of the nine regions was edited using Bioedit software, version 7.2.5 (Ibis Biosciences, Carlsbad, CA) and was assembled in a concatenated sequence of 6916 bases (around 23% of the genome length).

**Table 1:** Primers used for amplification of sequencing fragments

Amplification fragment	Target gene	Primer name, position* and sense	Primer sequence (5′–3′)	Expected size (bp)	References
1	ORF1a	MERS-3047 (+)	ACT GCG TGG AAT GCC GAT TC	843	This study
		MERS-3890 (−)	GCC TAC GAC ATG CAG GAT ATT C		
2	ORF1a	MERS-8089 (+)	CAA CAT TCA TTG ACG CAG CAC	946	[35]
		MERS-9034 (−)	GGA TCA TGG CAG TAT GGT GTC		
3	ORF1a	MERS-11419 (+)	CAA GCC CCA TTG CCT ATC TG	646	[34]
		MERS-12064 (−)	GCT TGA AGT ACG CTA GGA GTG		
4	ORF1b	MERS-18791 (+)	CAT CAA GGA GCT CAT GTG GC	792	[35]
		MERS-19582 (−)	TTC CAA ACC TTG AAC TTT TGT AAA AG		
5	Spike	MERS-21425 (+)	CTG TCG CAG GGT AAG TTA CTT ATC	756	This study
		MERS-22180 (−)	GTG TAC ATA AAG GTG CAG TTA CG		
6	Spike	MERS-22116 (+)	CGT AAT GCC AGT CTG AAC TC	918	[34]
		MERS-23032 (−)	CAG GGT GAG TAT TGA TTA GCG		
7	Spike	MERS-22990 (+)	CTG AAG TAC CTC AGT TAG TGA ACG	1225	This study
		MERS-24214 (−)	GCT GAT GCT GGA CCT TGC TG		
8	ORF3/4a	MERS-25465 (+)	GTG ATA GAT ACG AGG AAT ACG ACC	780	This study
		MERS-26001 (−)	GGA TAG CTG ACA GTT CCA CAG		
9	ORF4b	MERS-26042 (+)	CTT TGG CCA AAC AGG ACG CA	536	[34]
		MERS-26856 (−)	GAC GCC GAG AAA GCC ATA GTT C		

ORF = open reading frame, MERS = Middle East Respiratory Syndrome, bp = base pair

^*^Positions are relevant to the complete genome sequence of Human betacoronavirus 2c EMC/2012 (GenBank accession number JX869059)

### Sequence and phylogenetic analysis

The complete genome sequence of 92 MERS-CoV Saudi and international strains (67 of human-origin and 25 of camel-origin) were retrieved from the GenBank database. Strains were chosen to represent the different MERS-CoV genotypes [29, 30], the variable geographic regions worldwide, and the entire period of virus spread (2012 to 2019). Sequences were edited and trimmed using Editseq program of Lasergene software, version 3.18 (DNAStar, Madison, WI) to display concatenated sequences corresponding to those generated in the current study. Multiple sequence alignment for divergence analysis, identification of mutation sites, and prediction of amino acid substitutions was performed using Clustal W algorithm, MegAlign program, Lasergene v3.18. Heterogeneity in the glycosylation profiles of all MERS-CoV stains was assessed by determining the potential N- and O-linked glycosylation sites using Net-N-glyc 1.0 (http://www.cbs.dtu.dk/ services/NetNGlyc) and Net-O-glyc 3.1 (http://www.cbs. dtu.dk/services/NetOGlyc), respectively [31]. The phylogenetic tree was constructed based on both 2670 nts of the S gene. Phylogenetic analysis was accomplished using the maximum likelihood (ML) method of MEGA 6.0 software with bootstrapping of 1000 pseudo-replicates.

### Analysis of selective pressure

To investigate the evolutionary dynamics, identify and allocate statistically significant positive and negative selective pressure sites, codons over the entire concatenated sequence were screened using DataMonkey server (http://www.datamonkey.org) [32, 33]. Four different models were employed for such purpose including: (1) single likelihood ancestor counting (SLAC), which uses a combination of ML and counting approaches to determine dN/dS substitution rates per-site basis for coding sequence alignment and its phylogeny, (2) fixed effects likelihood (FEL), (3) random effects likelihood (REL), (4), mixed effects model of evolution (MEME), which was applied to allocate positively selected sites among different clades within a phylogenetic tree. Sites with a statistical *p*-value of < 0.1 were considered under positive selection.

### Nucleotide sequence accession numbers

The nucleotide sequence of the different genome fragments of MERS-CoV strains analyzed in this study have been deposited in GenBank under the following accession numbers: KT624236—KT624239 (ORF1a/b), KT624240—KT624243 (Spike gene), KT624244—KT624247 (ORF3), and KT624248—KT62451 (ORF4b).

## Results

### Sequence analysis, mutation record and glycosylation profile

**Fig. 2:**
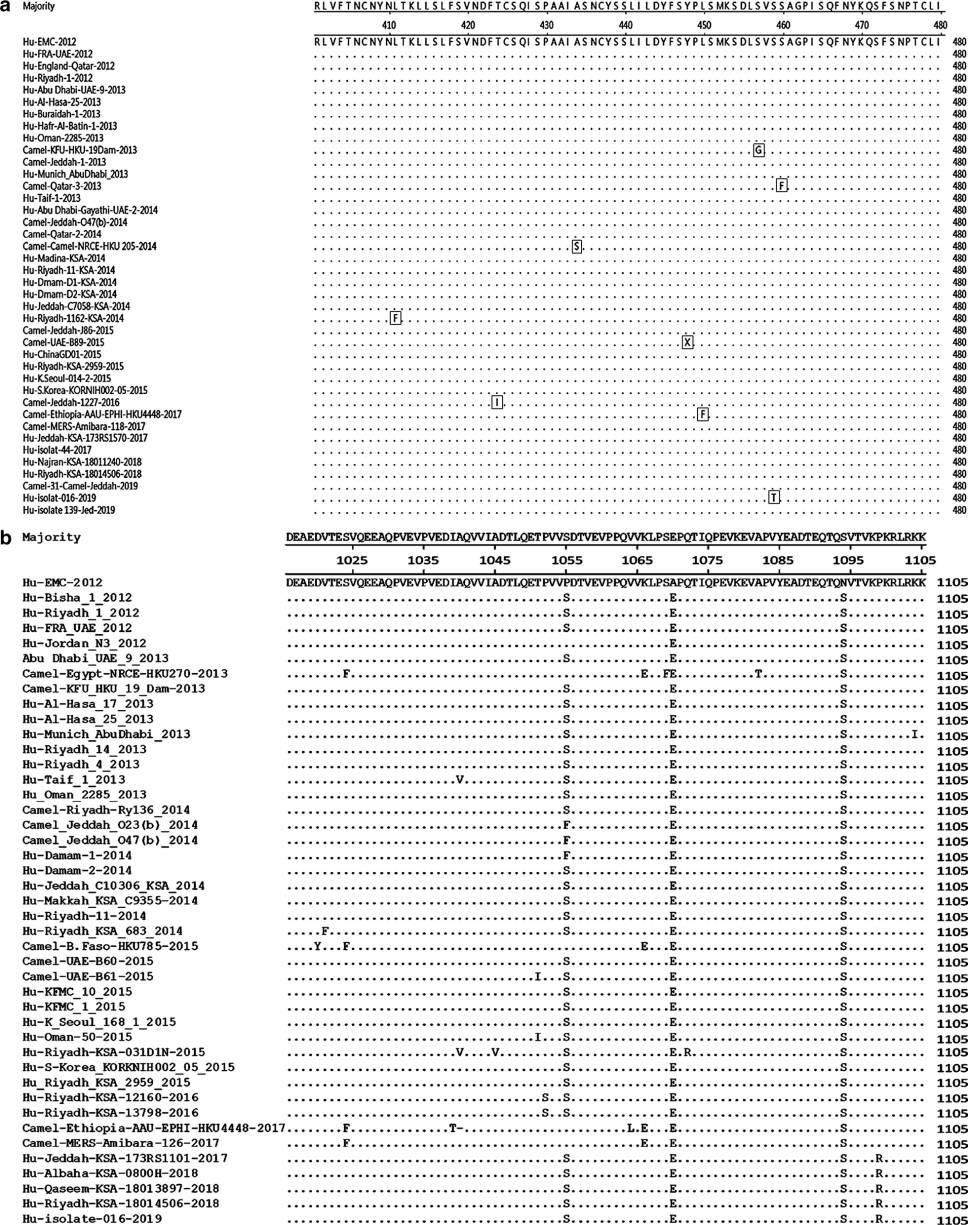
Deduced amino acid alignments of representative strains from different years were selected, and the alignment was done by Clustal W method running within the MegAlign program (DNAstar). Alignments are shown in comparison with the consensus sequences of the first isolated MERS-CoV strain (EMC-2012). Dots represent the identical amino acid residues. **a** displays 80 deduced amino acids of the S protein RBD. Host and year specific amino acids are shown in boxes. **b** 90 amino acid residues of ORF1a/b (residues 1025 to 1105) showed a distinct pattern where several amino acids had been changed permanently in comparison with the isolate (EMC-2012); 1000 T/V, 1055 P/S, 1070 A/E, 1094 N/S

Multiple alignment of the assembled concatenated sequence of the four Saudi MERS-CoV strains revealed no abnormal sequence variations like gaps, insertions, and/or deletions. The overall nucleotide and deduced amino acid sequence homology ranged from 99.1 to 100% and from 98.8 to 100%, respectively. A total of 27 mutations were recognized, among which 7 mutations changed their corresponding amino acid residues (Table 2). The majority of mutations (n = 14) was identified in S protein gene. To track the evolution of S and ORF1a/b proteins throughout the years 2012–2019, the deduced amino acid sequences of all analyzed sequences were compared to EMC-2012; the first MERS-CoV isolate. The spike protein seemed to be conserved among all the tested strains with about 99% sequence homology. Some amino acid changes appear characteristic to specific sub-clusters including: V27A, G159Y, H194Y, S390F, L450F A597V, R626P, L745F for MERS-CoVs of Ethiopian camels (2017), V27L for Riyadh and Qaseem human isolates (2018), L411F for isolates of Riyadh (2014), A756Q, E666K and M696T for camel isolates of UAE and Egypt (2014). More importantly, the receptor binding domain (RBD) of the S1 protein subunit (residues 358 to 588) displayed amino acid changes in several MERS-CoV strains, such as K369I in Camel-Jeddah-O47(b)-2017, S390F in strains of Ethiopian camels, S457G in KFU-HKU-19dam-2013, S460F in Qatar-3–2013, A434S in Camel-Egypt-NRCE-HKU-205, Y447X in Camel-UAE-D1209-2015, and D509G in both Bisha-2012 and Riaydh-1–2012 strains (Fig. 2a).

**Table 2:** Mutation record analysis of MERS-CoV strains identified in the current study

Gene	Mutation	Amino acid change	Positive/negative selection	Saudi MERS-CoV strains			
				Hu-Dammam_1_2015	Hu-Dammam_2_2015	Hu-Madinah_4_2015	Hu-Riyadh_11_2014
ORF1a	C3442T	–	Positive	+	−	−	−
ORF1a	C8518T	A2840V	Positive	+	+	+	+
ORF1a	A8846G	–	Negative	−	−	+	−
ORF1a	C8953T	S2985F	–	−	−	+	−
ORF1b	C19075A	–	Negative	+	+	+	+
Spike	T21569C	–	–	+	−	−	−
Spike	T21713C	–	–	−	+	+	+
Spike	C22169T	–	–	−	−	−	+
Spike	T22337C	–	–	+	−	−	−
Spike	C22349T	–	Negative	+	−	+	−
Spike	G22427T	–	–	+	−	−	−
Spike	T22895C	–	Negative	+	−	−	−
Spike	T23406C	–	–	+	−	−	−
Spike	T23504C	–	–	−	+	+	+
Spike	C23570T	–	Negative	−	+	+	+
Spike	C23648T	–	Negative	−	+	+	+
Spike	T23714G	–	–	+	−	−	−
Spike	C23756T	–	Negative	−	+	+	+
Spike	C23804T	–	–	−	+	+	+
ORF3	C25580T	L8527F	–	−	+	+	+
ORF3	C25656T	A8552V	–	+	−	−	−
ORF3	A25768T	–	Negative	−	+	+	+
ORF3	C25788T	S8596L	Positive	−	+	+	+
ORF4+b	T26109C	S8703T	–	−	+	+	+
ORF4b	G26167C	M8723T	–	+	−	−	−
ORF4b	C26216T	–	–	−	+	−	−
ORF4b	C26518T	–	–	+	−	−	−

ORF1a/b showed a distinct pattern where several amino acids had been changed permanently in comparison with the isolate (EMC-2012); 1000 T/V, 1055 P/S, 1070 A/E, 1094 N/S, 2747 A/V and 2780 A/V (Fig. 2b). Two amino acid changes, 981 G/S, and 1099 P/R, were found to be characteristic to the isolates of 2018 and 2019. Similarly, isolates of 2016 have one characteristic amino acid change at 1052 P/S. Four mutations were recorded in ORF3/4a and 4 in ORF4b (Table 2). Of these, 3 mutations changed the corresponding amino acids in ORF3/4a; L8527F, A8552V, and S8596L and two in ORF4b; S8703T and M8723T. Both N and O glycosylation sites were determined for the S protein. A total of 13 potential N-glycosylation sites, 66, 104, 125, 166, 222, 236, 244, 410, 487, 592, 619, 719, and 870, were reported for all the tested strains including our strains. Only one strain, isolate 1390-Hu-Jed, isolated in March of 2019 in Jeddah displayed an additional N-glycosylation site at 155. In the contrast, potential sites for O-glycosylation ranged from 2 to 4. In most strains, residues 135, and 878 seemed to be conserved for O-glycosylation.

### Codons under selective pressure

The adaptive evolution of MERS-CoV strains was evaluated by calculating dN/dS ratio (ѡ) for the entire amplified fragments. Most codons of the two major clades (A and B) displayed purifying selection with ѡ < 1 (0.329 for clade A and 0.438 for clade B). Only 5 codons, 4 in ORF1a (926, 1055, 2747 and 3785), and 1 in spike gene (7085) were found to have ѡ > 1 by the four different models, SLAC, FEL, REL and MEME with statistical significance values.

### Phylogenetic analysis

**Fig. 3:**
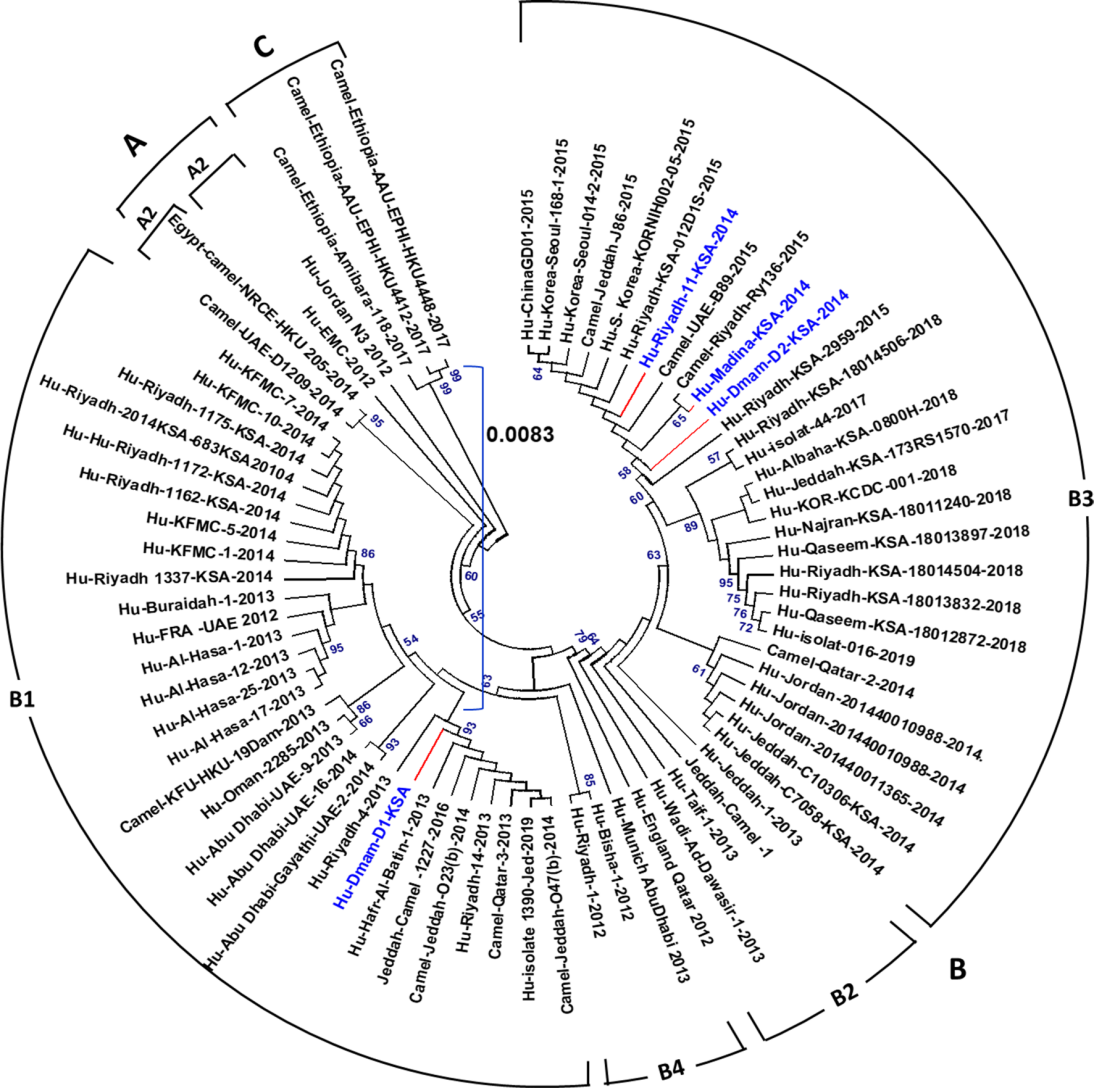
Phylogenetic tree based on the first 2670 nt of the S gene using MEGA 6.06 program. Multiple sequence alignment was performed using Clustal W, and the phylogram was generated by the Maximum Likelihood method based on the Tamura-Nei model. The analysis involved 73 nucleotide sequences. All positions containing gaps and missing data were eliminated. Only bootstrap values exceeding 50% are displayed. Clades and lineages are indicated at the periphery of the phylogram

The phylogenetic tree was constructed based solely on the nucleotide sequence of the spike gene. MERS-CoV strains were clustered into three main clades, A, B and C (Fig. 3). Clade A has four strains including the first strain isolate from Bisha, Saudi Arabia in 2012, an early strain from Jordan, and two camel strains from UAE and Egypt. The majority of MERS-CoV strains are grouped into clade B which is further divided into 4 distinctive lineages B1 to B4. Most of the Saudi strains, including those of the current study, are members of lineage B3. Strains from Korea and China are also grouped into lineage B3, whereas UAE strains of both human and camel origin are grouped in lineage B1. Clade C contains only three strains of camel origin isolated in Ethiopia in 2017.

## Discussion

MERS-CoV continues to circulate in the Middle East region causing sporadic cases, particularly in Saudi Arabia. The extinction of SARS-CoV-1, the causative agent of 2002–2003′s pandemic, the low spread rate of MERS-CoV, and the emergence of the current SARS-CoV-2 pandemic has raised several questions regarding coronavirus (CoV) genetics and evolution. In the current study, we aimed at analyzing the sequence of four MERS-CoV strains identified in the winter season of 2014–2015. These sequences were compared to a wide range of strains identified in both human and camel over 7 years (2012–2019). The use of concatenated sequences particularly those of the most important genes was found to be reliable for sequence and phylogenetic analysis of MERS-CoV [34, 35].

One of the interesting features of CoVs is its ability to use different cellular receptors. For instance, HCoV-229E uses Aminopeptidase N [36], SARS-CoV-1 and SARS-CoV-2 use angiotensin-converting enzyme 2 (ACE2) [37], whereas S protein of MERS-CoV binds to dipeptidyl peptidase 4 (DPP4) [20]. The location of S protein on the surface of virus envelope and its role in initiating virus infection makes it a preferred target for host defense and consequently a major hot spot for mutation. In comparison to EMC-2012, the first identified MERS-CoV strain, several characteristic amino acid changes were reported along the S gene including the RBD. These changes appear to be host and year specific. The host specificity of these amino acid changes in RBD may reflect adaptation to human or camel cellular receptors. Whereas year specific changes reflect *continuous accumulation of mutations* over time to coup with the immune response, only one codon of the S gene was found to be under selective pressure. In contrast, RSV and influenza viruses evolve mainly by introducing several point mutations in the attachment and hemagglutinin proteins, respectively. This finding adds more evidence that CoVs evolve by introducing major changes in the S gene through recombination rather than accumulation of point mutations. Glycosylation of viral glycoproteins usually modulate virus infectivity and antigenicity [38, 39]. Both N- and O-linked oligosaccharides may mask epitopes in certain cases and hence enables the virus to escape the immune system [40, 41]. In a previous study, we have reported that the variation in the glycosylation pattern of Saudi HRSV-A strains had a deep impact on virus infectivity and immunogenicity [42]. Here, we report a total of 13 potential N-glycosylation and from 2 to 4 O-linked glycosylation sites in the spike protein. These glycosylation sites may enhance virus infectivity and help to evade the pre-existing immunity.The drawback of the present study is the limited number of specimens used for analysis. Although a few number of (partial) sequence has been used, the data are still relevant and indicative. Even though the whole genome sequence is variable, however, the fragments included in the study are the most variable and therefore more suitable for studying virus evolution overtime. From our bioinformatic analysis of the entire MERS-CoV genome, we do not expect *a significant* difference in the results.

The relationship between different strains of MERS-CoV at the level of geographical distribution and host range (i.e. camel or human) was further elucidated in phylogenetic analysis. Although we used partial sequence of S gene (2670 nt), the tree topology was almost similar to that constructed using complete S gene and full genome sequences [43–46]. Because of the high sequence similarity between MERS-CoV strains of camel and human origins, clades displayed a mix of both. In a previous study, Lau et al., has classified the MERS-CoV strains into two major clades, A and B [43]. In this study, inclusion of MERS-CoV strains isolated from Ethiopian camels has resulted in appearance of a third clade (C). Clade C was recognized before to contain isolates from Ethiopia, Burkina Faso, Egypt, Morocco, and Nigeria, and therefore, it may be considered as an African clade [44].

MERS-CoV is largely circulating among dromedary camels in the Middle East region and in North and East Africa as evidenced by the high seropositive rates in many of the affected countries [47, 48]. However, human cases were not reported in most of the African countries and remained confined only to the Middle East region. This may be explained by the antigenic variation between the S protein of MERS-CoV isolated from dromedaries in Ethiopia and those isolated from the Middle East. Another explanation is that MERS-CoV strains of Ethiopian camels replicate at lower rates in tissue culture and are easily neutralized by lower concentration of sera [44].

Lineage B3 comprises most strains isolated in Saudi Arabia as well as in South Korea and China [49]. The virus was reportedly transmitted to South Korea via a Korean who performed multiple visits to the Middle East countries including Saudi Arabia. Interestingly, the same strain was transmitted to China via two infected persons who travelled from South Korea [50].

Accessory proteins play an important role in the pathogenesis of MERS-CoV. For example, the product of ORF4a inhibits interferon production through binding to dsRNA while the product of ORF4b mediate virus evasion of IFN actions [51, 52]. Therefore, amino acid changes in both ORFs may modulate the host immune response against MERS-CoV. Here, we reported four mutations in each ORF with amino acid changes in the corresponding codons. However, the impact of these amino acid changes on virus pathogenicity requires further studies.

## Conclusion

In conclusion, sequence and phylogenetic analysis of MERS-CoV overtime from 2012 to 2019 revealed the continuous evolution of the virus. MERS-CoV evolution was apparent in both ORF1a/b and the S gene. Interestingly, amino acid changes seemed to be host and year specific reflecting virus adaptation to host cellular receptors and evolution overtime to coup with the host immunity. Taking into consideration the inconsistent epidemiological patterns of HCoVs, we recommend frequent monitoring the sequences of MERS-CoV isolates particularly the S protein and understand these changes with regard to disease severity. Deep understanding of virus evolution will be useful to contain and develop effective tools for controlling any possible MERSCoV outbreak at early stages.

## Data Availability

Samples were collected and handled by the relevant authorities at the Saudi Ministry of Health (MOH-SA) following the strict biosafety guidelines set by WHO and CDC [7, 27]. Samples were transported in viral transport medium to the regional laboratories of MOH-SA and processed immediately or stored at − 80 °C till use.

## Abbreviations

MERS-CoV: Middle East Respiratory Syndrome-related Coronavirus
CoVs: Coronaviruses
